# Effects of a front-of-package disclosure on accuracy in assessing children’s drink ingredients: two randomised controlled experiments with US caregivers of young children

**DOI:** 10.1017/S1368980023001969

**Published:** 2023-12

**Authors:** Frances Fleming-Milici, Haley Gershman, Jennifer Pomeranz, Jennifer L Harris

**Affiliations:** 1 Rudd Center for Food Policy and Health, University of Connecticut, Hartford, CT 06103, USA; 2 Department of Public Health Policy and Management, School of Global Public Health, New York University, New York, USA

**Keywords:** Front-of-package labelling, Front-of-package disclosure, Sugary drinks, Fruit drinks

## Abstract

**Objective:**

Test effects of a standardised front-of-package (FOP) disclosure statement (indicating added sugar, non-nutritive sweetener (NNS) and juice content) on accuracy in assessing ingredients and perceived healthfulness of children’s drinks.

**Design:**

In two randomised controlled experiments, the same participants viewed drink packages and indicated if products contained added sugar or NNS and percent juice and rated drink healthfulness. Experiment 1 (E1) included novel (non-US) children’s drinks with a) product claims only (control), b) claims and disclosure, or c) disclosure only. Experiment 2 (E2) included existing children’s drinks (with claims) with a) no disclosure (control) or b) disclosure. Both experiments evaluated sweetened (fruit drink and flavoured water) and unsweetened (100 % juice and juice/water blend) drinks. Potential individual differences (education level and race/ethnicity) in effects were explored.

**Setting:**

Online survey

**Participants:**

Six hundred and forty-eight US caregivers of young children (1–5 years)

**Results:**

FOP disclosures significantly increased accuracy for most ingredients and drink types, including identifying presence or absence of NNS in sweetened drinks, no added sugar in juice/water blends, and actual percent juice in fruit drinks and juice/water blends in both experiments. Disclosures also increased recognition that the novel 100 % juice and juice/water blend did not contain NNS or added sugar (E1) and existing sweetened drinks contained added sugar (E2). Disclosures reduced perceived healthfulness of sweetened drinks but did not increase unsweetened drink healthfulness ratings. Some differences by participant socio-demographic characteristics require additional research.

**Conclusions:**

FOP disclosures on children’s drink packages can increase caregivers’ understanding of product ingredients and aid in selecting healthier children’s drinks.

Ages 1 to 5 years is a critical time for developing taste preferences for nutritious foods and healthy eating habits that support children’s optimal development and lifelong health outcomes. Therefore, experts recommend against providing any type of drink that contains added sugar and/or non-nutritive sweeteners (NNS)^(1–4)^. Providing sweetened drinks can reinforce a child’s natural taste preference for sweet and reduce acceptance of plain milk and water^(5)^, the only drinks recommended for children under the age of 6 years^(4)^. Despite these recommendations, 45 % of 2- to 4-year-olds in the USA consume sugary drinks on a given day^(6)^, primarily in the form of fruit drinks^(7)^, and 13 % of young children (2–5 years) consume drinks with NNS^(8)^. Further, children in households with less-educated (*v*. higher-educated) parents are more likely to consume sugary drinks^(9)^, and as compared with non-Hispanic White and Mexican-American children, Black children have higher rates of fruit drink consumption^(10)^.

Common sweetened children’s fruit-flavoured drinks in the US market, including fruit drinks and flavoured waters (labelled as ‘water’ beverages), contain added sugar and/or NNS with little or no juice^(11)^. Common unsweetened children’s drinks include 100 % juice and juice/water blends, consisting of juice diluted with water and no added sweeteners^(11)^. In 2018, US sales of sweetened children’s drinks totalled $1·4 billion, compared with $838 million for unsweetened children’s drinks^(11)^. Also, although companies spent more in total advertising for unsweetened *v*. sweetened drink products ($34·4 *v*. $20·7 million)^(11)^, they devoted a higher proportion of advertising spending in children’s media to sweetened drinks (66 % *v*. 54 %).

Parents purchase sweetened fruit-flavoured children’s drinks for many reasons, including marketing appeals, convenience, price, child requests, misperceptions about healthfulness and confusion with 100 % juice^(12–15)^. Research shows that common product packaging features contribute to this confusion^(15,16)^. For example, sweetened fruit-flavoured drink packages often include words such as ‘juice’, ‘nectar’ or fruit names^(17)^ and pictures of fruit, even when they contain little or no juice^(11,18)^. They also often contain nutrition-related marketing claims such as ‘100 % Vitamin C’ and ‘all natural’ ingredients, including ‘natural’ sweeteners (e.g. stevia, cane sugar)^(13)^. Parents report that these package features lead them to believe that sweetened fruit-flavoured drinks are healthy choices for their children^(15)^ and may not consider them to be ‘sugary drinks’ that are not recommended for children^(12,13,19,20)^.

Research has also shown that parents have difficulty distinguishing between sweetened and unsweetened options and identifying ingredients in drinks they serve their children^(15)^. Children’s drink brands often offer both sweetened and unsweetened drinks with similar branding and packaging features^(11)^, and parents have reported mistakenly purchasing sweetened drinks, believing they were 100 % juice^(15)^. In one study^(16)^, a majority of caregivers could not identify children’s drinks with NNS, and many incorrectly believed that unsweetened drinks contained added sugar, sweetened flavoured waters had no added sugar, and 100 % juice contained less than 100 % juice, even when looking at nutrition and ingredient information. Although parents express concerns about artificial sweeteners^(19,21,22)^, most could not identify NNS in the drinks they serve their children^(13)^. Further, individuals with a high school education or less are less likely to understand current ingredient information provided on pre-packaged food (i.e. nutrition facts panel)^(23)^; therefore, confusion about children’s drink ingredients may be greater among less-educated caregivers. Increasing parents’ knowledge of NNS, added sugar and juice content in children’s drinks may reduce misperceptions about the healthfulness of sweetened drinks^(13,24)^, which is associated with frequency of serving these drinks to their children^(12,19)^.

Therefore, efforts to increase caregivers’ understanding of ingredients in the drinks they serve their children may help reduce sugary drink consumption among young children. Previous research has demonstrated that adding warning labels and/or removing claims and fruit images from drink packages may also be effective^(20,24–26)^. However, government-mandated sugary beverage warnings (i.e. on billboards) have been successfully challenged in court and restrictions on truthful claims and images may not be legally feasible in the USA due to US constitutional protections for commercial speech^(18,27)^. Thus, experts have proposed front-of-package (FOP) disclosures that provide the same factual, uncontroversial and evidence-based information about all products in a standardised format to help consumers identify ingredients in both sweetened and unsweetened drinks and to reduce perceived healthfulness of sweetened children’s drinks^(16,28)^. However, to-date research has not examined the effectiveness of FOP disclosures that contain factual ingredient-specific information without warning labels or other interpretive information, nor the effects of NNS disclosures on product packages, and further whether disclosures are effective among caregivers of diverse race/ethnicity and education level.

This study tests the effects of a proposed standardised factual FOP disclosure on accuracy in identifying added sugar, NNS and percentage of juice in common types of children’s drinks and perceived product healthfulness^(20)^. We hypothesised that: H1) the disclosure would improve accuracy on both novel and existing sweetened and unsweetened children’s drink products; H2) accuracy would be greater if common claims were also removed; and H3) the disclosure would reduce perceived healthfulness of sweetened drinks. Exploratory analyses assessed whether the FOP disclosure also affected caregivers’ intent to purchase the products and whether effects of FOP disclosures were consistent across participant education level and race/ethnicity to ensure effectiveness for priority populations^(29)^.

## Methods

We conducted one online study with two randomised controlled experiments using the same participants in May 2022. To test H1, Experiment 1 (E1) examined the effects of FOP disclosures on accuracy of assessing added sugar, NNS and percent juice in novel (i.e. non-US) children’s drink products; and Experiment 2 (E2) examined effects on existing packages of a popular US children’s drink brand. In both experiments, participants viewed four package images: two sweetened drinks (fruit drink and flavoured water) and two unsweetened drinks (100 % juice and juice/water blend), as these are the four common types of children’s drinks available in the USA.^(11)^ In E1, participants were randomly assigned to one of three conditions: a) control (children’s drink packages with product claims), b) disclosure with claims (added to control packages) or c) disclosure only (with claims removed). In E2, they were assigned to one of two conditions: a) control (existing product package with claims) or b) disclosures (added to existing package). To test H2, we examined differences between the two disclosure conditions in E1 (disclosure with claims (b) and disclosure only (c)). To test H3, we examined effects of disclosures on perceived healthfulness of sweetened and unsweetened drinks in both experiments. Study procedures, hypotheses and analysis plan were registered prior to data collection on AsPredicted.org (https://aspredicted.org/Y9X_FMK). Data were analysed from October to December 2022.

### Participants

A national online survey panel company (InnovateMR) invited eligible panel members (US caregivers with children 1–5 years) to participate via email and sent a link to the study survey to interested participants. The survey was administered through Qualtrics software. Panel members received points for participating, which can be exchanged for online gift cards^(30)^. A target sample size of 600 was pre-determined based on power analysis assuming a medium effect size^(31)^. Quota sampling ensured at least 150 Black, 150 Hispanic and 150 participants with a high school education or less to evaluate differences by race/ethnicity and education. Prior to starting the survey, participants read an information sheet and indicated agreement to participate. Participants were then screened for eligibility (having a child 1–5 years, some responsibility for what that child eats and drinks, and no children with a disease/condition requiring a special diet). The University’s Institutional Review Board approved the study and determined it to be exempt as only survey data with no identifiable information were collected.

### Stimuli

**Fig. 1:**
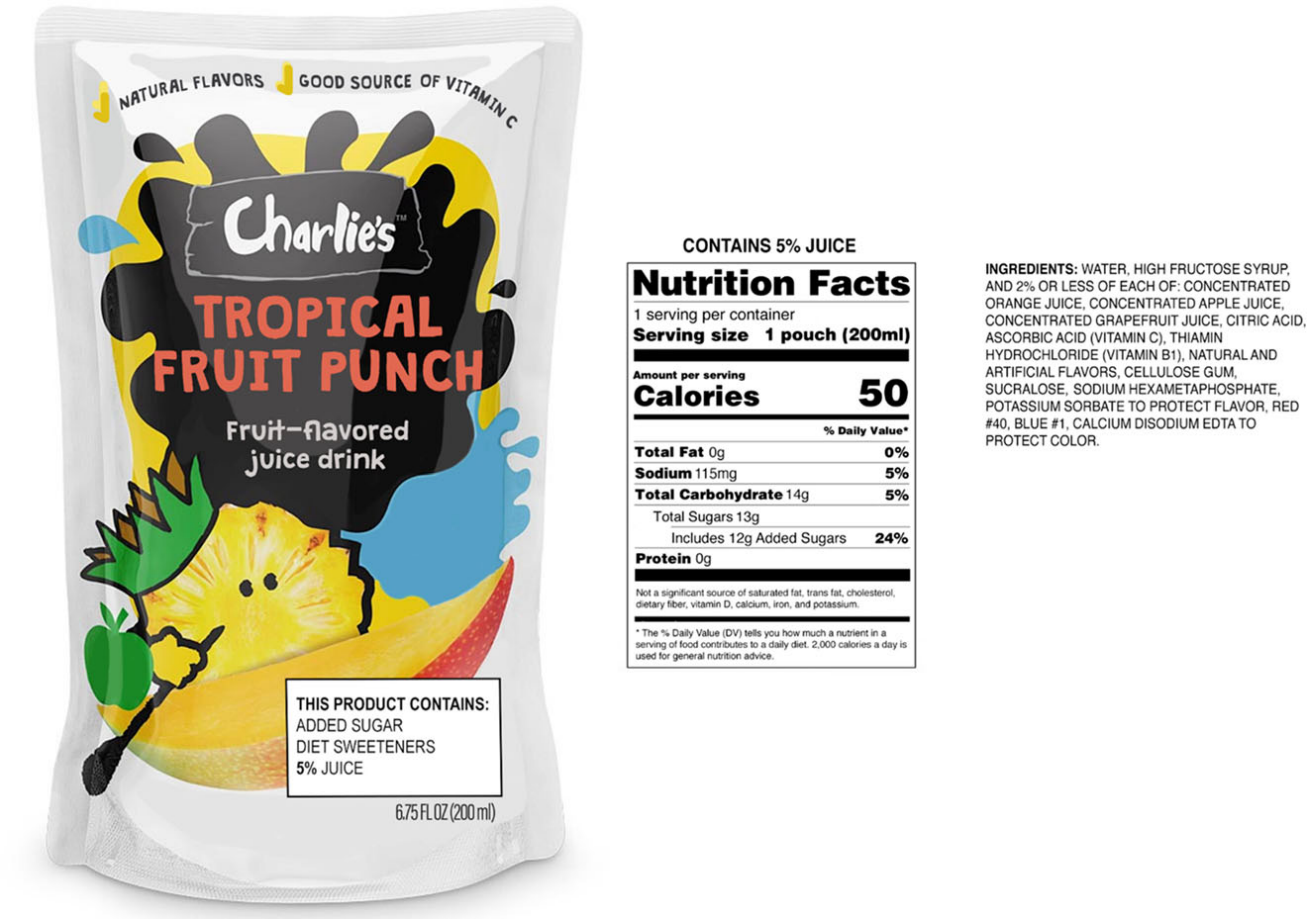
Novel fruit drink image with claims and FOP disclosure*. *Packages in all conditions contained the same nutrition facts and ingredient list for each product type. FOP, front-of-package

The packages to be used as stimuli were chosen following an online pre-test with caregivers of young children (1–5 years) (*n* 150). The pre-test assessed four alternative FOP disclosure designs and two alternative novel products not available in the USA (see online Supplementary Information for details). A graphic designer created the images for both experiments, which included the package front, nutrition facts panel (with percent juice at the top) and ingredient list (see Fig. 1 for one example and online Supplementary Information for all package images).

In both experiments, the ‘control’ condition (a) images incorporated information currently included on children’s drink packages. In the novel drink experiment (E1), we added two product claims that are commonly found on US drink packages (‘natural flavors’, ‘good source of Vitamin C’)^(11)^ and used the information panel (i.e. the nutrition facts panel, ingredient list and percent juice) from popular US children’s drinks in each drink category. Consistent with US regulations, the designation of a drink as 100 % juice was shown on both the package front and information panel. For the existing drink experiment (E2), the control condition utilised images of actual (Capri Sun) packages for each drink type, including claims and other product information on the existing package.

For both experiments, the ‘disclosure with claims’ condition (b) utilised the same packages as the control condition with the FOP disclosure box added. The FOP disclosure was configured to be less than 10 % of the entire front panel. The term ‘diet sweeteners’ was used to indicate NNS content on the FOP disclosure as previous research had ascertained that the term was widely understood and encompassed all types of NNS (including both ‘artificial’ and ‘natural’ high-intensity sweeteners)^(15,32)^. In E1, the ‘disclosure only’ condition (c) included the FOP disclosure on the condition (b) package, with the claims removed. Table 1 provides ingredient information and disclosures for all products in both experiments.

**Table 1:** Product ingredients, disclosures and claims

	Product ingredients					
Experiment/product	Added sugar	NNS	Juice content	Accurate response range	FOP disclosure	Claims
E1. Novel drinks (Charlie’s)						
Fruit drink	Yes	Yes	5 %	0–20 %	THIS PRODUCT CONTAINS: ADDED SUGAR DIET SWEETENERS 5 % JUICE	‘Natural flavors’ ‘Good source of vitamin C’
Flavoured water	Yes	Yes	0 %	0–20 %	THIS PRODUCT CONTAINS: ADDED SUGAR DIET SWEETENERS 0 % JUICE	‘Natural flavors’ ‘Good source of vitamin C’
100 % juice	No	No	100 %	90–100 %	THIS PRODUCT CONTAINS: NO ADDED SUGAR NO DIET SWEETENERS 100 % JUICE	‘All natural’ ‘Good source of vitamin C’
Juice/water blend	No	No	35 %	25–40 %	THIS PRODUCT CONTAINS: NO ADDED SUGAR NO DIET SWEETENERS 35 % JUICE	‘All natural’ ‘Good source of vitamin C’
E2. Existing drinks (Capri Sun)						
Fruit drink	Yes	No	10 %	0–20 %	THIS PRODUCT CONTAINS: ADDED SUGAR NO DIET SWEETENERS 10 % JUICE	‘35 % less sugar’ ‘All natural ingredients’ ‘NO high fructose corn syrup, artificial colors, flavors or preservatives’
Flavoured water	Yes	Yes	0 %	0–20 %	THIS PRODUCT CONTAINS: ADDED SUGAR DIET SWEETENERS 0 % JUICE	‘50 % less sugar’ ‘All natural ingredients’ ‘NO high fructose corn syrup, artificial colors, flavors or preservatives’
100 % juice	No	No	100 %	90–100 %	THIS PRODUCT CONTAINS: NO ADDED SUGAR NO DIET SWEETENERS 100 % JUICE	‘3/4 cup fruit juice’ ‘All natural ingredients’ ‘NO added sugar, artificial colors, flavors or preservatives’
Juice/water blend	No	No	56 %	45–60 %	THIS PRODUCT CONTAINS: NO ADDED SUGAR NO DIET SWEETENERS 56 % JUICE	‘All natural ingredients’ ‘NO added sugar, artificial colors, flavors or preservatives’

NNS, non-nutritive sweetener; FOP, front-of-package.

### Measures and survey design

After providing consent and answering eligibility questions, eligible participants were first randomised to one of three conditions in E1. They were informed that they would view some packages of children’s drinks that are not currently available in the USA but might be sold in the future and asked to look at the package and then answer questions about the drink. After viewing and assessing the four novel drink products (one for each drink type), they were then randomly assigned to one of the two E2 conditions. They were informed that they would see packages of children’s drink products available in the USA and asked to answer the same questions about these drinks. For both experiments, packages of the four drink types were presented in random order.

To assess participants’ accuracy in recognising drink ingredients, while viewing each package they were asked to indicate whether the drink contained added sugar (yes or no), diet sweeteners (yes or no) and percent juice in the product (0–100 % sliding scale). After rating all products in both experiments, they were shown the same eight novel and existing products previously viewed (presented in random order) and asked to indicate how likely they would be to buy each (for novel products, ‘if it was available in the US’) for their child(ren) who are 1–5 years old (sliding scale: 1 (extremely unlikely) to 10 (extremely likely)). They were also asked to rate how healthy they think the product is for their child (sliding scale: 1 (extremely unhealthy) to 10 (extremely healthy)).

After answering all questions in both experiments, participants were then asked about their provision of fruit drinks, flavoured waters, 100 % juice and juice/water blends to their child(ren) who are 1–5 years old (seven-point scales: ‘never’ to ‘3 or more times per day’) and if they had provided each type of Capri Sun in the past month (yes or no). Next, participants indicated their provision of other types of drinks to their child(ren) (toddler milk, plain milk, flavoured milk, plain water and other sugar-sweetened drinks), how often they themselves drink sugar-sweetened drinks, and knowledge and agreement with healthy beverage recommendations (data not reported). Finally, they provided demographic information.

### Statistical analysis

We coded accuracy according to whether participants correctly identified the presence or absence of added sugar and diet sweeteners. For percent juice, we conservatively coded responses as accurate if participants selected a percentage within a specified range, as in previous research^(16)^(see Table 1). We assessed significant differences between the control and disclosure conditions in the percent of participants who accurately identified added sugar, NNS and percent juice for each drink type and experiment. We used Kruskal–Wallis analyses for E1 (three conditions) and chi-square analyses for E2 (two conditions). *Post hoc* chi-square analyses examined the effects of removing claims on packages in E1 by comparing accuracy for all ingredients and drink types between the two disclosure conditions (with and without claims). In analyses to test effects of adding the disclosure on perceived healthfulness, we calculated average healthfulness ratings for sweetened drinks (fruit drink and flavoured water) and unsweetened drinks (100 % juice and juice/water blend) as the dependent variables and experimental condition as the independent variable, using multivariate ANOVA (MANOVA) for E1 and *t* tests for E2. Significance of all primary analyses was assessed using Bonferroni–Holm corrections to adjust for multiple comparisons within each experiment (*P* = 0·05, adjusted for fourteen comparisons).

Exploratory secondary analyses examined effects of disclosures on purchase intentions using MANOVA (E1) and *t* tests (E2), with purchase intention as the dependent variable and condition as the independent variable. To explore potential effects of disclosures on participants of differing race/ethnicity and education level, differences in accuracy by condition were also assessed separately for each individual characteristic using Kruskal–Wallis (E1) and chi-square (E2) analyses. Additional MANOVA assessed main effects of condition controlling for individual characteristics (education and race/ethnicity) on perceived healthfulness of sweetened and unsweetened drinks. For all exploratory analyses, we report significant differences at *P* < 0·05.

## Results

Of 1075 panel members who responded to the survey invitation, 17 declined participation, 304 did not meet eligibility criteria, and 106 did not complete the survey, finished the survey too quickly (< 5 min) or failed the data integrity check. See online Supplementary Information for CONSORT participant flow diagram. The final sample (*n* 648) was 73 % female and socio-demographically diverse, including 72 % with less than a 4-year college degree, 27 % Hispanic, 25 % non-Hispanic Black, and more than one-third with annual household incomes below $35 000 (see Table 2). The majority reported serving their child each type of children’s drink at least once in the past week, ranging from 66 % for flavoured water to 91 % for 100 % juice. More than one-half reported serving Capri Sun 100 % juice and/or fruit drink. Participants were equally divided into conditions for both experiments. Comparisons of demographic characteristics by condition demonstrated successful random assignment for both experiments (all *p*’s ≥ 0·07), as well as distribution of E1 condition by condition in E2 (*P* = 0·63).

**Table 2:** Sample characteristics

			Difference by condition	
Characteristic	No.	Perc	Novel drinks (E1)	Existing drinks (E2)
			*P*-value	*P*-value
Total sample	648	100 %		
Female	475	73·3	0·21	0·41
Age (years)			0·56	0·66
18–25	75	11·6		
25–34	328	50·7		
35+	244	37·7		
Education			0·19	0·99
High school or less	255	39·4		
Some college	209	32·3		
4-year degree or higher	184	28·4		
Race/ethnicity			0·59	0·98
Non-Hispanic White	269	41·5		
Non-Hispanic Black	162	25·0		
Hispanic	176	27·2		
Non-Hispanic other race	41	6·3		
HH income			0·21	0·10
< $35k	231	38·4		
$35–74·9k	206	34·2		
$75k+	165	27·4		
SNAP participants	244	37·7	0·54	0·92
WIC participants	157	24·2	0·73	0·45
Children in the household				
Ages 6–11 years	314	48·5	0·90	0·70
Ages 12–17 years	206	31·8	0·82	0·87
Served children’s drinks at least once in past week				
Fruit drinks	503	77·6	0·86	0·27
Flavoured water	427	65·9	0·95	0·35
Juice/water blends	532	82·1	0·52	0·37
100 % juice	592	91·4	0·71	0·07
Served Capri Sun in the past month				
Original (fruit drink)	339	52·3	0·13	0·21
Roarin’ Waters (flavoured water)	227	35·0	0·07	0·94
100 % juice	367	56·6	0·16	0·25
Organic (juice/water blend)	240	37·0	0·60	0·76
Other	66	10·2	0·71	0·86

HH, Household; SNAP, Supplemental Nutrition Assistance Program; WIC, Women, Infants, and Children Program.

### Effects of front-of-package disclosures on accuracy in identifying drink ingredients

In the first experiment with novel drinks (E1), the majority of participants in the control condition (packages with claims and no disclosures) recognised that the fruit drink and flavoured water contained added sugar and the 100 % juice did not, but approximately two-thirds mistakenly believed the juice/water blend contained added sugar (see Table 3). The majority also accurately perceived that the 100 % juice and juice/water blend did not contain NNS, but fewer than one-half recognised that the fruit drink and flavoured water did contain NNS. Approximately one-half of participants understood that the fruit drink and flavoured water contained less than 20 % juice. On average, participants in the control condition believed that the fruit drink and flavoured water contained 30 % or more juice, although the products contained 5 % and 0 % juice, respectively. Just 42 % accurately identified the percent juice in the juice/water blend, and only 62 % correctly indicated that the 100 % juice contained 100 % juice.

**Table 3:** Effects of FOP disclosures on accuracy of assessing drink ingredients and product attitudes

	Novel drink experiment (E1)								Existing drink experiment (E2)					
	Control (claims only) (a)		Claims and disclosure (b)		Disclosure only (c)				Control (no disclosure) (a)		Claims and disclosure (b)			
Sample size	213		219		216				327		321			
Accuracy†	%	95 % CI	%	95 % CI	%	95 % CI	*P*-value	Accurate	%	95 % CI	%	95 % CI	*P*-value	Accurate
Added sugar														
Fruit drink	86·9	82, 91	87·2	82, 91	88·0	83, 92	0·940	Yes	78·9	74, 83	87·9	84, 91	0·002 *	Yes
Flavoured water	76·5	70, 82	84·0	79, 88	86·6	81, 91	0·018	Yes	75·5	71, 80	85·7	81, 89	0·001 *	Yes
100 % juice	56·8	50, 63	70·8	64, 76	75·0	69, 80	<0·001 *	No	70·6	65, 75	80·1	75, 84	0·005 *	No
Juice/water blend	36·6	30, 43	62·6	56, 69	69·4	63, 75	<0·001 *	No	65·7	60, 71	77·3	72, 82	0·001 *	No
NNS														
Fruit drink	47·9	41, 55	72·1	66, 78	72·7	66, 78	<0·001 *	Yes	65·1	60, 70	78·8	74, 83	<0·001 *	No
Flavoured water	44·1	38, 51	72·1	66, 78	76·9	71, 82	<0·001 *	Yes	45·3	40, 51	75·7	71, 80	<0·001 *	Yes
100 % juice	70·4	64, 76	78·5	73, 83	86·1	81, 90	<0·001 *	No	72·5	67, 77	81·3	77, 85	0·008 *	No
Juice/water blend	60·6	54, 67	74·0	68, 79	79·6	74, 84	0·005 *	No	69·4	64, 74	77·9	73, 82	0·015 *	No
Percent juice														
Fruit drink	49·8	43, 56	63·5	57, 70	66·7	60, 73	0·001 *	0–20 %	53·8	48, 59	67·6	62, 72	<0·001 *	0–20 %
Flavoured water	53·5	47, 60	64·4	58, 70	67·1	61, 73	0·009 *	0–20 %	56·9	51, 62	67·3	62, 72	0·006 *	0–20 %
100 % juice	62·4	56, 76	67·1	61, 73	70·4	64, 76	0·216	90–100 %	71·6	66, 76	74·1	69, 79	0·460	90–100 %
Juice/water blend	42·7	36, 49	57·1	50, 63	63·0	56, 69	<0·001 *	25–40 %	59·0	54, 64	75·4	70, 80	<0·001 *	45–60 %

Product attitudes‡	M	95 % CI	M	95 % CI	M	95 % CI	*P*-value	Eta sq	M	95 % CI	M	95 % CI	*P*-value	Cohen’s d
Perceived healthfulness (1–10)														
Sweetened drinks	5·71	5·34, 6·07	4·97	4·61, 5·33	4·61	4·24, 4·97	<0·001 *	0·028	5·90	5·63, 6·18	4·93	4·65, 5·21	<0·001 *	0·38
Unsweetened drinks	6·43	6·15, 6·70	6·82	6·55, 7·09	6·60	6·33, 6·88	0·134	0·006	7·08	6·86, 7·30	7·13	6·91, 7·35	0·745	0·02
Intent to purchase (1–10)														
Fruit drink	5·65	5·25, 6·05	4·98	4·58, 5·37	4·88	4·48, 5·28	0·015	0·013	6·84	6·53, 7·16	5·84	5·53, 6·16	<0·001 *	0·35
Flavoured water	5·62	5·22, 6·01	4·62	4·23, 5·01	4·43	4·04, 4·82	<0·001 *	0·030	6·43	6·09, 6·76	5·21	4·87, 5·54	<0·001 *	0·40
100 % juice	7·49	7·14, 7·83	7·63	7·29, 7·97	7·52	7·18, 7·87	0·846	0·001	7·98	7·73, 8·23	8·33	8·08, 8·58	0·052	0·15
Juice/water blend	5·73	5·36, 6·09	5·81	5·44, 6·16	5·80	5·45, 6·17	0·946	0·000	7·05	6·77, 7·33	6·68	6·40, 6·97	0·073	0·14

FOP, front-of-package; NNS, non-nutritive sweetener.

* Significant after Bonferroni–Holm correction (*P* = 0·05 adjusted for fourteen comparisons).

† Accuracy assessed as follows: proportion of participants who selected the accurate response. Significant differences between conditions assessed using Kruskal–Wallis (E1) and chi-square (E2).

‡ Differences between conditions in product attitudes assessed using multivariate ANOVA (MANOVA) (E1) and *t* tests (E2).

The effects of FOP disclosures on accuracy in identifying ingredients in novel drinks (E1) varied by ingredient and drink type. There were significant differences in the proportion of participants who recognised that the 100 % juice and juice/water blend product did not contain added sugar; the fruit drink and flavoured water contained NNS and the 100 % juice and juice/water blend did not; and accurate assessment of juice in the fruit drink, flavoured water and juice/water blend. In all cases, accuracy was higher in the two disclosure conditions than the control, but there were no significant differences between the two disclosure conditions (all *P*’s ≥ 0·04) (data not reported).

In E2 with existing products, two-thirds or fewer of participants who viewed the actual product package without the disclosure (control condition) recognised that the juice/water blend did not contain added sugar, the fruit drink did not contain NNS, and the accurate percent juice in the fruit drink and flavoured water. Accuracy was lowest for knowing that the flavoured water contained NNS (45 %). Most participants (≥ 70 %) understood that the fruit drink and flavoured water contained added sugar, the 100 % juice did not contain added sugar or NNS, and the percent juice in 100 % juice. Adding FOP disclosures to existing drinks significantly improved accuracy for all drinks and ingredients with one exception: it did not increase understanding that the 100 % juice contained 100 % juice.

The disclosures reduced perceived healthfulness of sweetened drinks in both experiments, with small to medium effect sizes, and perceived healthfulness did not differ significantly between the two FOP disclosure conditions in E1. However, disclosures did not affect perceived healthfulness of unsweetened drinks in either experiment. In addition, disclosures reduced intent to purchase the sweetened fruit drink and flavoured water in both experiments but did not affect purchase intent for unsweetened drinks.

### Exploring differences by education level and race/ethnicity

Results of the exploratory analyses to examine whether FOP disclosures affected participants of differing education level and race/ethnicity were inconsistent (see online Supplementary Tables S1 and S2). In four cases, disclosures increased accuracy across all education levels and race/ethnicities: understanding that the novel juice/water blend did not contain added sugar, and identifying NNS in the three drinks that contained them (novel fruit drink and both flavoured waters). In most other analyses, we found significant differences for some but not all individual characteristics. However, disclosures did not consistently affect one group *v*. the others. In many cases, disclosures increased accuracy for individuals with a high school education or less but not for those with a 4-year college degree or higher, and for non-Hispanic Black or Hispanic individuals and not those in other racial/ethnic groups. In contrast, the main effects of disclosures on perceived healthfulness of sweetened drinks remained significant and effects on perceived healthfulness of unsweetened drinks remained non-significant in the models with race/ethnicity and education level as independent variables (see online Supplementary Table 3).

## Discussion

These results largely support our first hypothesis (H1) that FOP disclosures improve caregivers’ ability to assess added sugar, NNS and percent juice in both novel and existing children’s drink products. In both experiments, disclosures increased accuracy for twenty of twenty-four ingredients disclosed (i.e. eight different drinks with three ingredients disclosed on each), including identifying absence of added sugar in juice and juice/water blends (in both experiments) and presence of added sugar in Capri Sun fruit drinks and flavoured water; presence or absence of NNS in sweetened drinks and absence of NNS in 100 % juice and juice/water blends; and actual percent juice in fruit drinks, flavoured water and juice/water blends. When effects of disclosures were non-significant, accuracy was relatively high (i.e. > 60 %) in the control condition with no disclosures, including recognising added sugar in novel sweetened drinks and percent juice in 100 % juice (both experiments).

Therefore, the disclosures provide an opportunity to increase caregivers’ understanding and awareness of ingredients in children’s drinks that raise major public health concerns^(1,4)^. Although awareness of sugar in novel fruit drinks and flavoured waters was relatively high without disclosures, the disclosures increased awareness that Capri Sun fruit drink and flavoured water contain added sugar, products that most participants reported giving to their child. Disclosures also increased understanding that some children’s fruit drinks and flavoured waters contain NNS, an ingredient most parents do not believe is safe for their children’s consumption^(33)^ but have difficulty recognising when disclosed only in ingredient lists on nutrition facts panels^(16)^. Disclosures were also effective at increasing understanding that healthier juice/water blends do not contain added sugar and contain moderate amounts of juice.

These findings are similar to studies that have examined effects of warning labels. FOP warnings, including symbols to highlight ‘high-in’ ingredients to avoid or statements about health harms related to consumption, have been found to correct misperceptions about the healthfulness of fruit drinks and reduce selection for their children^(24–26)^. The results assessing caregivers’ base-level understanding of ingredients in children’s sweetened and unsweetened drinks when viewing packages without disclosures (i.e. control conditions in both experiments) are also in line with previous research showing limited knowledge that fruit drinks and flavoured waters often contain NNS and misunderstandings about added sugar content in flavoured waters and unsweetened juices^(16)^.

Our results did not support H2. Contrary to predictions, accuracy did not improve when common claims were removed from novel drink packages that contained disclosures. Previous research has shown that removing claims on fruit drinks can increase adults’ accurate understanding of ingredients^(20)^, decrease perceived healthfulness^(20,26)^, and reduce intent to purchase for their children^(20)^and consume themselves^(26)^. However, research in this area is somewhat inconsistent. One study found that removing claims did not reduce parents’ selection of sweetened fruit drinks for their child if a fruit image remained on the package^(24)^. The same study showed that either adding warnings or removing both claims and fruit imagery on product packages reduced parents’ selection of sugar-sweetened fruit drinks for their child but did not test a condition with warnings added and claims removed^(24)^. Research is needed to understand the relative contribution of adding warnings or disclosures and/or removing claims and imagery in reducing parents’ purchases of sweetened drinks for their children.

Moreover, the present research shows that even when claims and fruit imagery were present on product packages, the disclosure corrected misperceptions about ingredients and healthfulness of sweetened drinks. Similar to our findings, previous research shows that warning labels were effective in reducing parents’ sugary drink selection for their children when added to packages that also contained fruit imagery and marketing claims^(24)^. Therefore, FOP disclosures alone may provide enough information to counteract common claims, such as ‘good source of Vitamin C’, ‘all natural ingredients’ and ‘no high fructose corn syrup’, that may lead caregivers to believe that sweetened children’s drinks are healthful choices for children^(20)^.

Our results supported H3: FOP disclosures reduced perceived healthfulness of sweetened drinks for both novel and existing products. However, they did not increase perceived healthfulness of unsweetened drinks. Similarly, in the exploratory analyses, disclosures reduced caregivers’ intentions to purchase both types of sweetened drinks (fruit drinks and flavoured waters) for their child but did not increase intent to purchase unsweetened drinks. The disclosure also reduced perceived healthfulness and purchase intentions for sweetened drinks regardless of the presence of claims on product packages. As perceived healthfulness is strongly associated with parents’ purchases of sweetened drinks for their children^(12,19)^, this finding indicates that disclosures could be an effective tool to reduce purchases of sweetened children’s drinks in addition to increasing understanding of drink ingredients. However, further research is needed to test this hypothesis. Additional research is also needed to determine whether disclosures lead caregivers to substitute 100 % juice or juice/water blends for sweetened children’s drinks and/or affect provision of plain milk or water, as recommended by public health experts^(4,34)^.

Exploratory analyses found that disclosures did not consistently affect all socio-demographic groups when results were stratified by education level and race/ethnicity. However, we found no evidence that the disclosures were consistently less effective with less-educated (*v*. higher-educated) caregivers or, Black or Hispanic (*v*. non-Hispanic White) participants. In many cases, differences were significant for these groups and not for more highly educated and/or non-Hispanic White participants. In addition, the disclosures continued to reduce perceived healthfulness of sweetened drinks when race/ethnicity and education level were included in the models. These findings help to address concerns raised by advocates that FOP changes must be appropriate for all consumers^(29)^, but future research is needed to examine effects of disclosures with larger samples of individuals in populations of concern (i.e. lower education level, Black and Hispanic).

### Strengths and limitations

This study has a number of strengths. To our knowledge, it is the first to assess disclosures of NNS on children’s drink packages, and the first to examine and demonstrate effects of a single standardised disclosure that indicates the presence or absence of NNS and added sugar and the percent juice. The randomised controlled design demonstrates causal impact of adding the FOP disclosure, including on packages of novel products and existing drinks that were widely purchased by our participants, even on sweetened drink packages with nutrition-related claims.

This study also has limitations. Use of quota sampling ensured a diverse sample, but findings are not representative of the US population. The randomised controlled design demonstrates causal impact of adding FOP disclosures in an online setting when participants were asked to examine the packages. However, these findings may differ in a real-world situation such as the supermarket where purchase decisions occur under time constraints and with competing brands and drink categories with various package claims and stocked on shelves together. Further, asking participants to identify product ingredients before rating product healthfulness likely affected absolute healthfulness ratings. However, participants answered the same questions in the same order in all conditions, allowing us to conclude that any differences in perceived healthfulness between conditions were due to the different stimuli they viewed (i.e. presence or absence of FOP disclosure). It is also possible that whether or not participants saw disclosures in E1 affected their accuracy in E2. However, E1 conditions were randomly distributed by condition in E2, and the effects of the disclosures remained significant in all models after controlling for E1 condition (analyses not reported). In addition, although we found no evidence that FOP disclosures were consistently more or less effective with individuals of differing education level or race/ethnicity, future research with larger sample sizes is needed to determine potential differential effects among individuals of diverse socio-demographic characteristics. Finally, our findings suggest potential effects on purchase intentions. Behavioural intentions (i.e. purchase intent) have been shown to predict dietary behaviours^(35)^, and previous research shows that perceived healthfulness is associated with parents serving sweetened children’s drinks^(12,19)^, but future research is needed to determine the effects of the FOP disclosure on actual purchasing behaviour.

### Policy implications

These findings can help inform efforts to develop FOP information to help consumers identify healthy foods when grocery shopping, as outlined in the US White House Strategy for Hunger, Nutrition and Health^(36)^. The federal government should act to address inadequacies of current labelling, by requiring the disclosures tested in this study on the FOP of children’s sweetened and unsweetened drinks. In the USA, the FDA regulates beverage labels and has the authority to revise certain labelling requirements on beverages that contain or purport to contain fruit juice (i.e. fruit-flavoured sweetened drinks and unsweetened juices) to address misleading labelling practices; however, the FDA frequently acts only when Congress expressly requires it to do so. Further, some labelling changes (e.g. requiring the percent juice on the FOP) require an act of Congress^(18)^. The First Amendment of the US Constitution protects commercial speech, which includes product labelling, from government interference. However, the government routinely requires factual disclosures on products to provide information and prevent deception of consumers. The tested disclosures meet constitutional requirements for government-mandated disclosures on product labelling; they are purely factual and uncontroversial, evidence-based (justified), about the products at issue, reasonably related to the government’s interest in preventing deception of consumers, and not unduly burdensome as they occupied less than 10 % of the FOP^(27,28)^.

The proposed factual disclosures may be more legally and politically feasible than warnings at this time. Warnings are a common feature of the consumer protection landscape, and a meta-analysis showed them to be an effective way to reduce sugary drink purchases^(25)^. A properly worded and formatted warning on products themselves should similarly survive First Amendment scrutiny^(37)^. However, the Court of Appeals for the Ninth Circuit found San Francisco’s billboard warning requirement for sugary beverages to be unconstitutional^(38,39)^. In striking down this warning, several judges made sweeping statements doubting the link between added sugar and health harms and inconceivably arguing that only warnings dating back to 1791 are constitutional^(37)^.

Certain claims may be deceptive or misleading, and these would be also ripe for regulation (e.g. ‘no artificial sweeteners’ when the product contains stevia extract)^(18)^; conversely, prohibiting factually accurate claims (e.g. ‘no high fructose corn syrup’) on product packaging is not a legally feasible policy option^(40)^. Another method to address misleading claims would be for the FDA to reconsider its fortification policy to disallow the fortification of drinks with added sugars. Currently, fruit drink labels tout high levels of Vitamin C due to fortification, which has been found to increase consumers’ perception that the drinks are healthy^(20)^. Prohibiting the fortification of fruit drinks would be a method to minimise potentially misleading vitamin claims on fruit drinks. Nonetheless, this study confirms that standardised FOP ingredient disclosures were effective at increasing consumer understanding even when common claims remained on the package. Therefore, requiring the proposed standardised FOP disclosure may be an effective path to reducing purchases of products not recommended for children’s consumption, including drinks with NNS and/or added sugar.

## Conclusion

Misperceptions about the ingredients and healthfulness of sweetened children’s drinks contribute to high levels of sugary drink consumption among young children. This research demonstrates that a clear standardised disclosure of added sugar and NNS and the percent of juice content on children’s drink packages provides enough information to correct these misperceptions and would aid parents in selecting healthier drinks for their children. Given other proposed FOP changes to reduce sugary drink consumption may not pass judicial scrutiny, the proposed disclosure may be both an effective strategy to reduce children’s sweetened drink consumption and a potentially viable policy change.

## Supplementary material

For supplementary material accompanying this paper visit https://doi.org/10.1017/S1368980023001969
